# *Listeria Monocytogenes* Brain Abscesses in a Patient with Disseminated Non-Small Cellular Lung Cancer: MRI Findings

**DOI:** 10.3390/diagnostics11061115

**Published:** 2021-06-18

**Authors:** Anders Lykkemark Simonsen, Nitesh Shekhrajka, Frederik Boëtius Hertz, Jannik Helweg-Larsen, Åse Bengård Andersen, Anne-Mette Lebech

**Affiliations:** 1 Heart Centre, Department of Infectious Diseases, Rigshospitalet, Blegdamsvej 9, DK-2100 Copenhagen Ø, Denmark; jannik.helweg-larsen@regionh.dk (J.H.-L.); aase.bengaard.andersen@regionh.dk (Å.B.A.); anne-mette.lebech@regionh.dk (A.-M.L.); 2 Department of Neuroradiology, Rigshospitalet, Blegdamsvej 9, DK-2100 Copenhagen Ø, Denmark; nitesh.shekhrajka@regionh.dk; 3 Department of Clinical Microbiology, Rigshospitalet, Blegdamsvej 9, DK-2100 Copenhagen Ø, Denmark; frederik.boetius.hertz@regionh.dk

**Keywords:** *Listeria monocytogenes*, neurolisteriosis, brain abscess, MRI

## Abstract

Brain abscesses caused by *Listeria monocytogenes* (LM) are very rare and carry a high mortality risk. We present a patient with disseminated non-small cellular lung cancer (NSCLC) and multiple unusual LM brain abscesses. These abscesses have multiple elongated peripherally enhancing lesions in a characteristic formation that is “worm or tramtrack-like” following the white matter fiber tracts.

A 39-year-old female diagnosed with disseminated non-small cellular lung cancer (NSCLC) was admitted after one day of developing malaise, fever (39.3 °C), and a reduced consciousness (Glasgow Coma Scale ≥ 13). This was also the day after third-line carboplatin/vinorelbine treatment. One year earlier, she was diagnosed with disseminated pulmonary adenocarcinoma, which had progressed despite intensive chemotherapy and radiation therapy. Upon admission, she received 25 mg of prednisolone daily (0,36 mg/kg). Laboratory testing revealed discrete signs of inflammation and anemia. White blood cells were 8.5 × 10^9^/L (normal range: 3.5–8.8 × 10^9^/L), neutrophilocytes were 7.2 × 10^9^/L (normal range: 1.6–5.9 × 10^9^/L), hemoglobin was 5.3 mmol/L (normal range 7.3–9.5 mmol/L), and C-reactive protein was 24 mg/L (normal range: 0–10 mg/L). After blood and urine culturing, empiric treatment with piperacillin/tazobactam was initiated because of suspected sepsis. On the second day, the fever and a headache continued. Three days after admission, although the fever and headache were resolved, she developed increasing confusion (Glasgow Coma Scale ≥ 13) and a discrete right-sided central facial nerve palsy. Blood cultures now grew *Listeria monocytogenes* (LM). In brief, we used the BACTEC^TM^ (Becton Dickinson, Franklin Lakes, NJ, USA) detection system. When the blood culture was positive, Gram staining and light microscopy were performed. Gram-positive rods were all identified via conventional culturing and cultured species were identified by the use of a matrix-assisted laser with desorption/ionization time-of-flight mass spectrometry (MALDI-TOF MS) (Bruker, Nordic AB, Kista, Sweden). The treatment was changed to high-dose ampicillin without gentamycin, which was contraindicated because of recent cisplatin treatment. The cerebrospinal fluid (CSF) analysis was normal, including normal CSF lactate at 2.2 mmol/L (normal range: 1.1–2.2 mmol/L), CSF-Protein at 0.5 g/L (normal range: 0.15–0.5 g/L), and CSF-Glucose at 3.3 mmol/L (normal range 2.2–3.9 mmol/L). CSF cultures and CSF PCR by the BioFire^®^ Filmarray^®^ PCR of CSF, which include a PCR for LM, were negative (BioFire Diagnostics, LLC, Salt Lake City, UT, USA, The FilmArray meningitis/encephalitis [ME] panel). The CSF was centrifuged and cultured. Cerebral MRI demonstrated multiple elongated peripherally enhancing lesions involving the right frontal lobe, the right half of the genu of corpus callosum, the right basal ganglia, and the right cerebral peduncle, which were tracked along the expected location of white matter tracts and U-fibers with subtle susceptibility artifacts and perifocal vasogenic edema. No lesions were observed in the left cerebral hemisphere or in the cerebellum. No lepto/pachymeningeal or cranial nerve enhancement were observed (Figure 1). These findings were compatible with CNS abscesses rather than metastasis, since involvement of the corpus callosum and predominantly white matter is atypical for the latter. Unilateral and unilobar involvement with elongated lesions following white matter tracts without lesions in the rest of the supra- or infratentorial brain or in the meningeal enhancement were considered less likely due to metastases.

After five days of treatment with ampicillin, two g q.4.h treatment was altered to meropenem two g t.i.d., to allow outpatient treatment. Intravenous antibiotics were continued for six weeks, with a plan of four weeks of subsequent oral antibiotic treatment.

After four weeks of treatment, an MRI demonstrated a complete resolution of contrast enhancement along the right frontal and basal ganglia lesions with partial resolution of surrounding vasogenic edema, but also an increase in susceptibility artifacts along the lesions (Figure 2). 

Due to severe nausea and vertigo after three days of amoxicillin/clavulanic acid 500 mg/125 mg t.i.d., the treatment was altered to moxifloxacin 400 mg once daily. This affected the liver with elevated ALAT 105 U/L (normal range: 10–45 U/L) and was discontinued after five days. Sulfamethoxazole/trimethoprim 1200/240 mg t.i.d. induced side-effects with severe nausea and vomiting. Twenty-three days after initiation of the first peroral regimen, an MRI demonstrated continued regression. Due to this and the side effects, it was decided to discontinue the treatment. Ten weeks after treatment discontinuation, the patient passed away due to her NSCLC, without showing any signs of recurrence of LM.

LM is an intracellular food-borne Gram-positive bacteria, which is widely distributed in nature. Risk factors for bacteriaemia and neurolisteriosis include high age, diabetes, and immunosuppression such as malignancy and organ transplantation with immunosuppressive therapy. CNS listeriosis may happen secondary to haematogenous dissemination or by retrograde axonal spread from the peripheral nervous system [1]. Animal studies have suggested that LM can reach the CNS via the cranial nerves by axonal intracellular spreading [2,3]. In a large French prospective cohort study on 252 patients with neurolisteriosis, meningoencephalitis was present in 84% of the cases and 17% had brain-stem involvement [4]. Brain abscesses are very rare and are only present in 1–10% of cases with neurolisteriosis [4,5]. Only 75 cases of CNS abscesses are reported in the literature between 1968 and 2017. The mortality rate was 27.3%, blood cultures were positive in 79.5%, and LM was isolated from the CSF or brain abscesses in 50.8% of reported cases [6]. Once in the CNS, animal studies have suggested further axonal intracellular spreading along white matter fiber tracts in the CNS [7,8]. A retrospective review of the literature found six cases of unilateral involvement of multiple abscesses, involving more segments of the brain and with accessible neuroimaging, all of which were distributed along white matter fiber tracts, supporting this theory [9]. An important differential diagnosis to be considered, especially in our case, was metastases, but unilateral and unilobar elongated lesions along the white matter tracts without lesions elsewhere in the brain or at grey-white junctions made metastases an unlikely diagnosis. LM brain abscess is a very rare disease though and may present without these characteristic radiologic findings. A large French prospective cohort study on 71 patients with neurolisteriosis did not demonstrate any significant neuroimaging pattern [10]. The fact that LM is more common in immunosuppressed patients may increase the incidence of neurolisteriosis and brain abscesses due to LM in the future [11,12]. In cases with negative blood cultures and where a lumbar puncture is contraindicated, multiple elongated peripherally enhancing lesions following the white matter fiber tracts in a “worm or tramtrack-like” appearance could be of great diagnostic value [6,9,13]. Two other diseases though show similar patterns of sparganosis and melioidosis, caused by *Spirometra mansoni* and *Burkholderia pseudomallei*, respectively, which are both mainly endemic in Southeast Asia [14]. Given the high mortality of LM brain abscesses, early diagnosis and treatment initiation is vital.

## Figures and Tables

**Figure 1:**
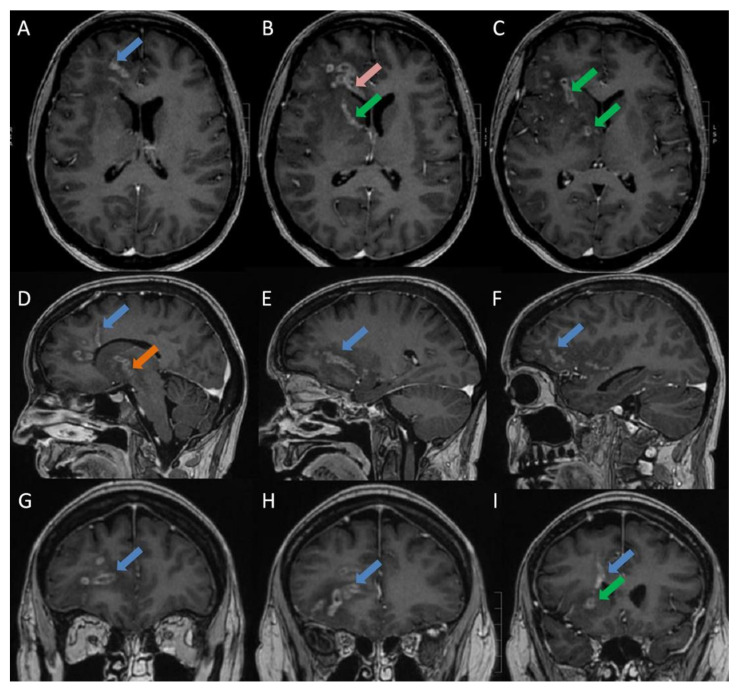
Initial MRI brain at admission: T1 BRAVO post contrast sequences—Axial (**A**–**C**: cranial to caudal), Sagittal (**D**–**F**: medial to lateral) and Coronal (**G**–**I**: anterior to posterior): shows multiple elongated peripherally enhancing lesions in the right frontal lobe (blue arrows), the right half of the genu of corpus callosum (pink arrow), the anterior limb of the right internal capsule (green arrows) as well as the right external capsule and insula, tracking along the expected location of white matter tracts and U- fibers also involving the right cerebral peduncle and right side of mesencephalon (orange arrow). No lesions were observed in the left cerebral hemisphere or in the cerebellum. No lepto- or pachymeningeal enhancement. No enhancement occurred along the cranial nerves.

**Figure 2:**
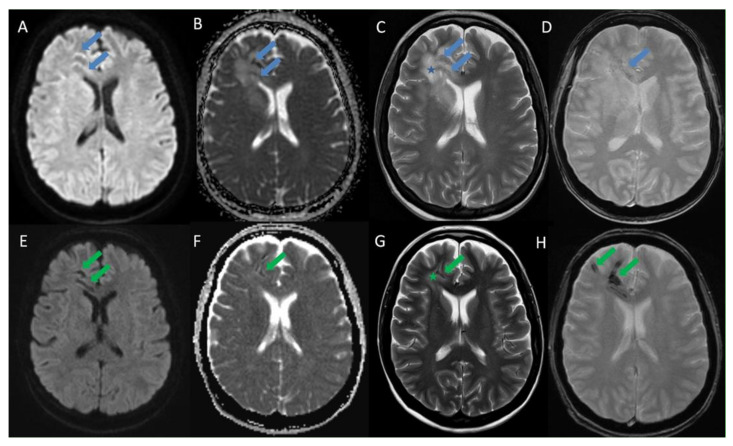
Initial MRI brain at admission (upper row) vs MRI brain 4 weeks after treatment (lower row) at the level of lateral ventricles: Axial DWI (**A** and **E**), Axial ADC (**B** and **F**), Axial T2 (**C** and **G**) and Axial T2* (**D** and **H**): The initial scan in the upper row shows no true restricted diffusion but prominent ADC hypointensities corresponding to the lesions which are thought to be secondary to subtle punctate foci of susceptibility artifacts/inflammatory free radicles (blue arrows). Surrounding vasogenic edema in the brain parenchyma is noted (blue star). The follow-up MRI 4 weeks after treatment shows an increase in the susceptibility artifacts blooming along the lesions on the T2* sequence with corresponding reduction of T2 signals and ADC values (green arrows). The surrounding vasogenic edema is also decreased (green star).
